# Transgenic HPV11-E2 protein modulates URR activity in vivo

**DOI:** 10.1007/s11248-023-00336-y

**Published:** 2023-02-24

**Authors:** Shubei Wang, Vera Gramm, Elke Laport, Tim Holland-Letz, Angel Alonso, Johannes Schenkel

**Affiliations:** 1 Cryopreservation W430, German Cancer Research Center, Heidelberg, Germany; 2 Institute for Physiology and Pathophysiology, University of Heidelberg, Heidelberg, Germany; 3 Biostatistics C060, German Cancer Research Center, Heidelberg, Germany; 4 Tumor Virology F050, German Cancer Research Center, Heidelberg, Germany; 5 Deutsches Krebsforschungszentrum (DKFZ) W430, Im Neuenheimer Feld 280, 69120 Heidelberg, Germany

**Keywords:** HPV-E2, Human papillomavirus type 11, Reporter gene, Transgenic mouse model, Upstream regulatory region URR

## Abstract

In vitro experiments have shown that the E2 protein of human papillomaviruses (HPV) binds to the upstream regulatory region (URR) of the viral genome and modulates transcription. Additionally, it seems to be a necessary component for viral DNA replication together with E1. We have developed a transgenic mouse model containing the URR region of the low-risk virus HPV11 that regulates the expression of the lacZ reporter gene. Most interestingly, in these mice, the transgene was exclusively expressed in the bulge region of the hair follicle but not in any other tissues. Further experimental data indicate that in double transgenic mice that also express the HPV11-E2 protein under the control of the Ubiquitin C-promoter, the transcription of the reporter gene is modulated. When E2 is present, the expression of the reporter gene also occurs exclusively in the bulge region of the hair follicles as it does in the single transgenic mice, but the expression of the lacZ driven by the URR is increased and the statistical spread is greater. Even if the expression of the reporter gene occurs in the hair follicles of the dorsal skin of an animal uniform, E2 obviously has the capacity for both to induce and to repress the URR activity in vivo.

## Introduction

Human papillomaviruses (HPV) are epitheliotropic small tumor viruses with a circular double-stranded DNA genome of ≈ 8 kb. More than 90 different HPV types have been identified so far, some of which are preferentially associated with benign lesions (low-risk types, e.g. types 1, 6, and 11) whereas high-risk types are found mainly in association with malignant tumors (e.g. types 16, 18, and 33). HPV11 is classified as a low-risk HPV and causes recurrent condyloma in the anogenital region and respiratory tract (Archambault and Melendy 2013). 90% of all genital warts are caused by HPV11 and HPV6 (zur Hausen 2008).

The upstream regulatory region (URR) of the viral genome is responsible for transcription of the putative oncogenes E6 and E7 as well as for the partially conserved open reading frames of the early proteins E1, E2 and the late genes L1 and L2. Among others Lin et al. (2002) or Sim et al. (2008) have demonstrated that transcription from the URR is regulated by the viral protein E2.

E2 is a 42 kDa, primarily nucleic phosphoprotein with a tripartite secondary structure: It has a C-terminal DNA binding and dimerization domain connected to the N-terminal transactivation domain by a flexible linker (Antson et al. 2000). The E2 protein is a transcription factor that binds to a specific recognition palindromic sequence ACCN6GGT as dimers and regulates the promoter sequences responsible for transcription of the E6 and E7 genes by binding to two sites located immediately upstream of the E6/E7 promoters. The mutation of these E2-binding sites disables the capacity to bind to E2 (Romanczuk et al. 1990).

The E2 protein can either activate or suppress transcription. Factors influencing that are E2 concentration, promotor type, cell type, position of E2-binding sites, nature and type of interactions of E2 polypeptides (McBride 2013; Bouvard et al. 1994; Steger and Corbach 1997). The transcription is initiated at the promoters located in the URR, the E6- and the E7 gene.

Several mouse models for human papillomaviruses have been described, for high-risk viruses (Cid et al. 1993) as well as low-risk viruses (Schenkel et al. 1999). A major concern of these approaches is that they are at least partially artificial, a human virus is investigated in another species and host or virus specific factors are not available.

We have developed an in vivo approach to analyze the effects of HPV11-E2 on the gene expression of the viral early genes. A double-transgenic mouse line was generated by crossing the HPV11-lacZ line with a mutant expressing HPV11-E2 ubiquitously under the control of the human Ubiquitin C-promoter (Leykauf et al. 2008). The presence of E2 leads to an upregulation of the number reporter of gene expressing hair follicles but not to an activation of the URR11 driven reporter gene in other tissues. However, the reporter gene expression is uniform and the genetic background of the animals seems to be important.

## Materials and methods

### Animals

The mutant mouse lines used in this study were B6;D2-Tg(HPV11-lacZ)1704Aal (short: URR11-lacZ; Schenkel et al. 1999) and B6;D2-Tg(UBC-HPV11E2)613Josc (short: UBC-HPV11E2; Leykauf et al. 2008), both on an unknown back-cross generation. They were housed in the animal facility of the German Cancer Research Center (DKFZ), Heidelberg, Germany. None of these GM lines developed a genotype related phenotype that was harmful. The animals were kept under standard housing conditions. The health of the animals was monitored according to the FELASA recommendations (Mähler et al. 2014). Animal experiments were performed according to the guidelines of the German Animal Welfare Act, approved by the Animal Welfare Department of the Competent Authority (Regierungspräsidium Karlsruhe, Germany), and conducted under the surveillance of the intramural Animal Welfare Committee.

Hemizygous mutated animals of each line were mated, resulting in mice carrying hemizygously either one or both mutations. All offspring were genotyped as described earlier (Schenkel et al. 1999; Leykauf et al. 2008). For back-crosses to the FVB/N genetic background, hemizygous mutated males were mated with FVB/N females and the offspring were also genotyped as shown above. Transgenic F1 back-cross males were selected for back-crossing with FVB/N females for the next generation.

For most experiments mice aged three to six months were investigated, to study a possible role of the age animals aged six to 18 months were used, too.

### X-Gal staining

Staining was carried out according to Schenkel et al. (1999) or Protopapa et al. (1999) with the beta-galactosidase reporter-gene staining kit (GALS, Sigma). Beta-galactosidase hydrolyzes 5-bromo-4-chloro-3-indolyl-β-D-galactopyranoside (X-Gal) into galactose and 5-bromo-4-chloro-3-hydroxyindole. The latter spontaneously dimerizes and is oxidized into 5,5'-dibromo-4,4'-dichloro-indigo, an insoluble blue dye reporting the expression of beta-galactosidase.

### Viewing of slides and data processing

Each X-Gal stained histology slide was scanned by a microscope (Zeiss, inverse, 1021955207), viewed at a (final) 100 × magnification and analyzed with the computer program TissueFACS (TissueGnostics, Vienna, Austria). To minimize error of analyses, each slide was divided into “fields of view” and these were then individually examined. The total number of hair follicles as well as the total number of positive follicles (dyed blue) were counted. There was no differentiation of the different hair cycle phases of the follicles at the time when the analysis was made. The numbers of total and positive hair follicles were obtained by adding the number from the three biopsies taken from each side of each mouse.

### Immunofluorescence

Sections were stored at − 20 °C. After thawing, they were dried for 10 min at 37 °C and 2 h at room temperature (RT), followed by circling with a „PapPen“ (a fatty pen to enclose the section). Next, 15 min fixation with 4% formaldehyde in phosphate buffered saline (PBS) at RT. Then, three washing steps with PBS + 0.05% Triton X-100 for 10 min, followed by blocking with blocking buffer (10% Goat-Serum, 0.3% Triton-X100 in PBS) for 2 h at RT.

The first antibody, anti-bacterial beta-galactosidase, was against the transgene (anti-*E. coli,* polyclonal chicken antibody, Abcam ab9361). This was diluted 1:800 (with 0.3% Triton-X100 1% BSA in PBS) and the slide was incubated overnight at 4 °C. The second antibody was added after three washing steps (with PBS + 0.05% Triton X-100 for 5 min). Depending on the experimental needs, either a green fluorescent (A-11039 Thermo Fisher Scientific Alexa Fluor 488; 1:1000) or a red fluorescent antibody (ab 97145 Abcam Cy3; 1:50) was added. The second antibody was diluted (with 0.3% Triton-X100 1% BSA in PBS) together with 4′, 6-Diamidin-2-phenylindol (DAPI, blue fluorescent). The final concentration of 2 µg/mL) for 60 min (A-11039) or DAPI was added in a separate step (ab 97145). Nuclei were stained by DAPI. Next, three washing steps with PBS were carried out for 5 min. Finally, the slides were covered with DAKO Faramount.

To analyze fluorescent dyes, confocal microscopy (Leica TCS SP5 II camera with software LAS-AF) was used. Fluorophores were excited by a laser λ = 358 nm (DAPI) λ = 488 nm (Alexa488) or λ = 552 nm (Cy3).

### Quantification

To quantify the expression of the reporter-gene chromaslides were used as standard. Chromaslides (Chroma Technology Corporation. 10 Imtec Lane, Bellows Falls, VT 05101, USA) are autofluorescent slides, frequently used to assess microscope settings. For quantification the fluorescence value of the chromaslide was determined. The fluorescence intensity of the tissue to be investigated was measured; the median was calculated and then divided through the chromaslide value. This led to normalized results.

### Western blots

The transgenic E2-expression was determined by Western blotting using standard procedures. 1 cm^2^ dorsal skin of mice was homogenized in 1% SDS containing proteinase inhibitors (Roche, Mannheim, Germany). The homogenate was spun at 10,000*g* for 30 min at room temperature and the supernatant was saved. Protein concentration was measured using the DC-protein assay system (Bio-Rad, Feldkirchen, Germany). Therefore, 1:1000 diluted triplets of all protein samples were prepared, the concentration were determined by using the absorption of Coomassie-blue. Eight differently concentrated albumin samples served as a standard, the concentration was detected in a microplate-reader.

For Western blotting, proteins were separated by SDS–polyacrylamide gel electrophoresis, blotted onto Polyvinylidene Fluoride (PVDF) membranes and reacted with the primary mouse-anti-human antibody ab100968 (Abcam). To demonstrate that similar amounts of proteins were loaded onto the gels, a parallel gel with the same amount of proteins was blotted and reacted with antibodies to b-actin. Following washing, the membranes were incubated with a peroxidase (POD)-labelled secondary antibody and reacting bands were revealed by chemiluminescence (ECL).

### Statistics

The statistical evaluation was carried out using the program GraphPad Prism 5. The unpaired t-test was used to compare two independent samples of different sizes from two normally distributed populations of equal variance. Samples were considered independent of each other if they came from two different populations, e.g. if the animals had different genetic backgrounds or were subjected to different applications at different times. Data point dependent samples, i.e. internal controls, were analyzed using a paired t-test. With the One-Way-ANOVA-test, three or more independent samples were compared in one characteristic. It assumes a normally distributed population of the samples as well as equal variances and corresponds to the unpaired t-test for a sample number > 2. The Mann Whitney U-test is a nonparametric test. It was used to compare the distribution of two independent samples where a normal distribution cannot be assumed. The F-test was used to show whether two samples from different, normally distributed populations differ in terms of their variance. Among other things, it also serves to check the equal variance assumption for unpaired t-tests and ANOVA-tests and its non-significance was a prerequisite for using either of these methods. In case of unequal variances, the Welch variant of these tests was used. Any differences were considered significant if *p* < 0.05.

## Results

### URR x E2 driven reporter-gene expression

**Fig. 1:**
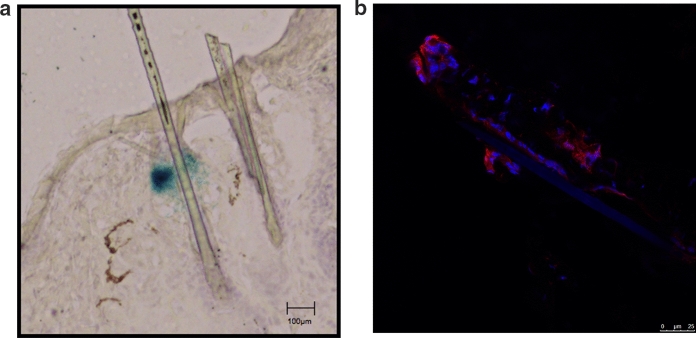
**a** Histochemical detection of beta-galactosidase: 6 µm cryosections of the dorsal skin of transgenic mice were prepared, fixed and stained with X-Gal. Slides were counterstained with hematoxylin/eosin. The blue color shows the reporter-gene expressing hair follicles (example). **b** Hair follicles stained with an anti-bacterial beta-galactosidase and a second antibody that shows a red fluorescence. Nuclei were counterstained with 4′, 6-Diamidin-2-phenylindol (DAPI, blue fluorescent). (Color figure online)

**Fig. 2:**
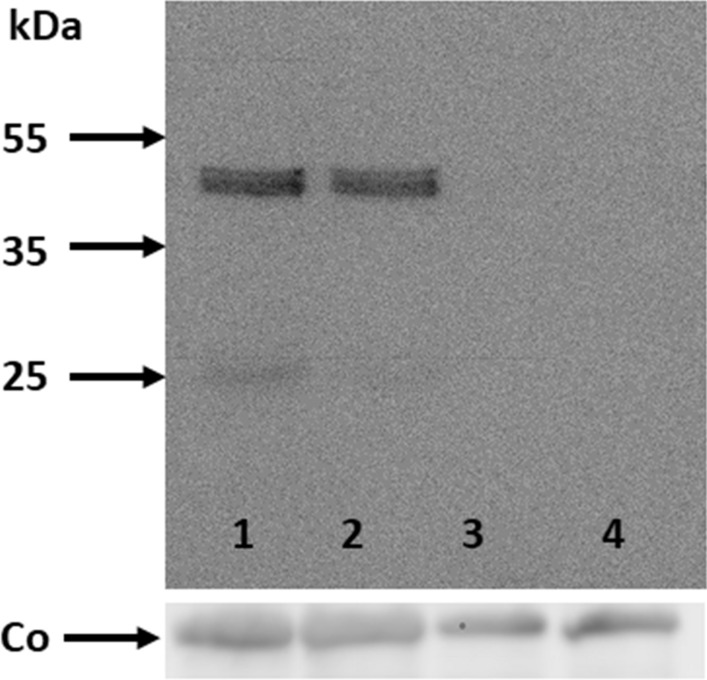
Transgenic E2 protein analysis in Western blots. The expression of the 42 kDa E2-protein was detected in the dorsal skin protein extract of four URR11-lacZ x UBC-HPV11E2 double transgenic mice (lanes 1, 2), whereas line 3 represents a URR11-lacZ single transgenic and lane 4 a wildtype mouse. “Co” represents the loading control (anti-b-actin)

To understand the role of the transgenic HPV11-E2-protein, URR11-lacZ x UBC-HPV11E2 double transgenic animals were screened for reporter-gene expression by using the approved X-Gal assay, i.e. employing the catalytic activity of the reporter-gene beta-galactosidase. Surprisingly, the reporter-gene was, as in presence of E2 (Schenkel et al. 1999), also only detectable in hair follicles of the dorsal skin (Fig. 1a) and not in other tissues. Therefore, the organs and other tissues shown to express the transgenic HPV11-E2-protein (Leykauf et al. 2008) were elucidated for the expression of the URR11 driven reporter-gene in the double-transgenic mice. To verify the transgenic HPV11-E2 expression, the availability of the protein was proven by performing Western blotting (Fig. 2). A 42 kDa band was detected in the protein extracts of the dorsal skin of URR11-lacZ x UBC-HPV11E2 double transgenic animals. URR11-lacZ and WT animals served as controls not expressing the transgenic E2-protein. This finding was a most interesting observation, since it clearly demonstrates that expression of the lacZ gene, under the control of the URR promotor, needs cellular factors present exclusively in epithelial cells of the host.

To further characterize this expression, we performed confocal microscopy using antibodies against the beta-Galactosidase protein. As shown in Fig. 1b, this demonstrated that the expression of the reporter gene is mainly localized at the hair follicle bulge and also on the cells located at the root of the hair. URR11 driven reporter gene expression was never detected in embryonic states or neonates.

**Fig. 3:**
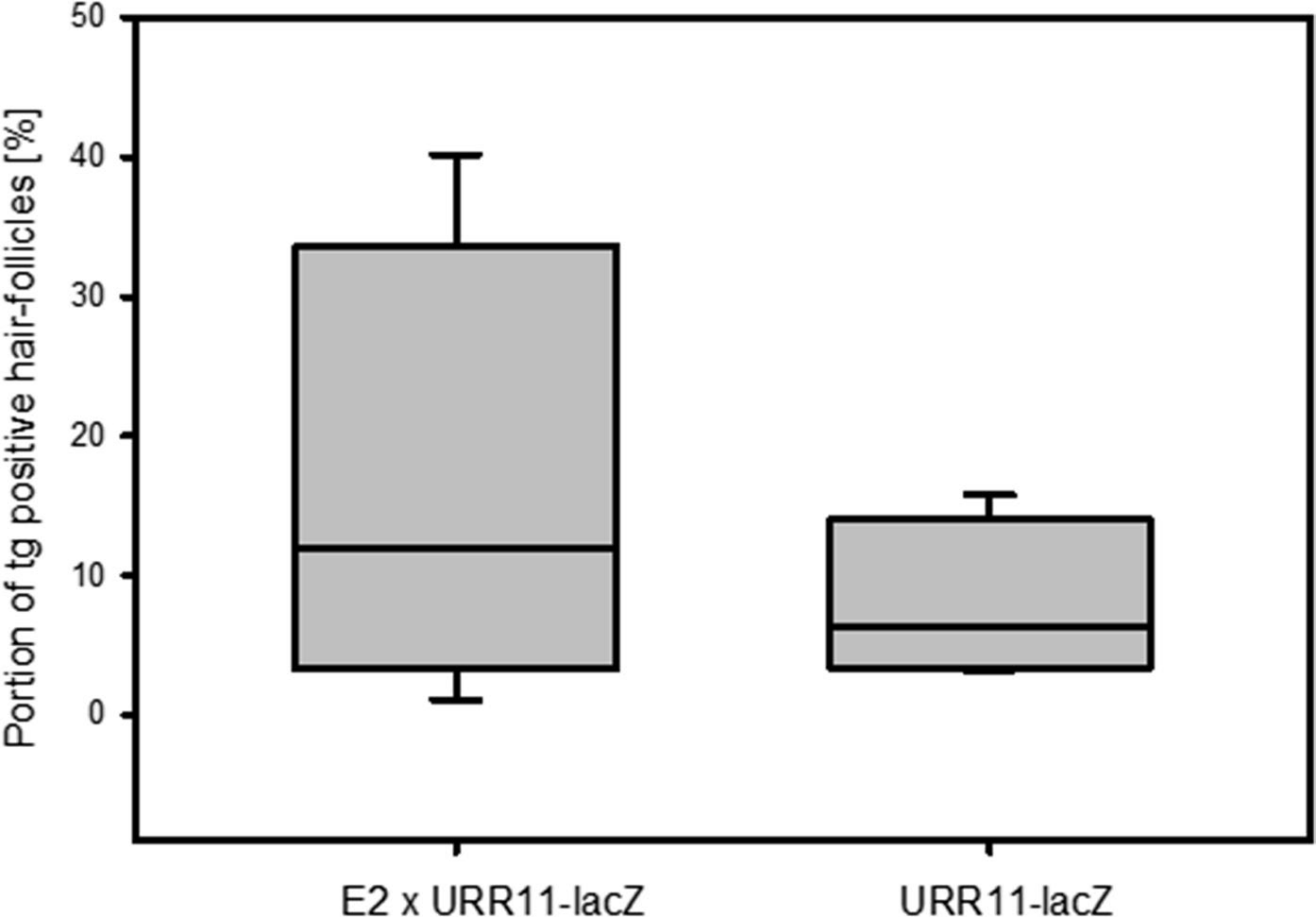
URR-11 activity in the presence and absence of the E2 protein. 11,527 hair follicles (out of these 1584 reporter-gene expressing) of 50 double transgenic mice were compared with 3832 hair follicles (out of these 322 reporter-gene expressing) of 14 single-transgenic URR11-lacZ mice. The presence of E2 resulted in a higher portion of reporter-gene positive hair follicles (13.74% vs 8.4%, *p* < 0.001, two-sided Welch-t-Test) and higher standard deviation (9.72% vs. 3.19%, *p* < 0.001, F-Test). The median was 13.45% vs. 8.45%

The influence of the transgenic E2-protein was studied in 11,527 hair follicles (out of these 1584 reporter-gene expressing) of 50 double transgenic mice and this was compared with 3832 hair follicles (out of these 322 reporter-gene expressing) of 14 single-transgenic URR11-lacZ mice. UBC-HPV11-E2 single-transgenic animals served as negative control (Fig. 3). The statistical analyses showed that the presence of E2 resulted in a higher portion of reporter-gene positive hair follicles (13.74% vs 8.4%, *p* < 0.001, two-sided t-Test), and higher standard deviation (9.72% vs. 3.19%, *p* < 0.001, F-Test). The median was 13.45% vs. 8.45%.

The hair follicles of different areas of the dorsal skin investigated exhibited the same pattern as described above. Mice aged three to six months were investigated in the studies described above. However, the age did not play a significant role in the activity of the transgenic URR in mice, though a slight trend to an upregulation of URR11-positive hair follicles with an age between 6 and 18 months of the animals was observed (data not shown in detail).

Attempts to induce URR-11 activity in the presence of the E2 protein using dexamethasone or UV-radiation treatment as described in a previous study in the absence of the transgenic E2-protein (Schenkel et al. 1999) resulted in an enormous statistical spread, subsequently without any significant differences (data not shown in detail).

### Effect of the genetic background on transgene expression

Woodworth et al. (2004) and Uc et al. (2020) have shown that the FVB/N genetic background might be suitable for studies on human papillomaviruses, as demonstrated by several mouse models with HPV16, as compared to the C57BL/6 or Balb/c genetic backgrounds. Therefore, the URR11-lacZ mice were back-crossed to the FVB/N genetic background. In the first and second back-cross generation an increase in the relative number of transgene-positive hair follicles was detected, however, the results did not show statistical significance (details not shown).

### Quantification of transgene expression

As shown above, the presence of E2 resulted in an overall high variance of the URR11-driven reporter-gene expression. These data are based on qualitative approaches. To better understand if the expression of the transgene is uniform, i.e. if the reporter-gene expression is up- or downregulated on different locations of the same animal or if the expression is rather uniform on a sample (e.g. on a slide), we tried to assess the quantity with a relative simple approach using chromaslides.

**Fig. 4:**
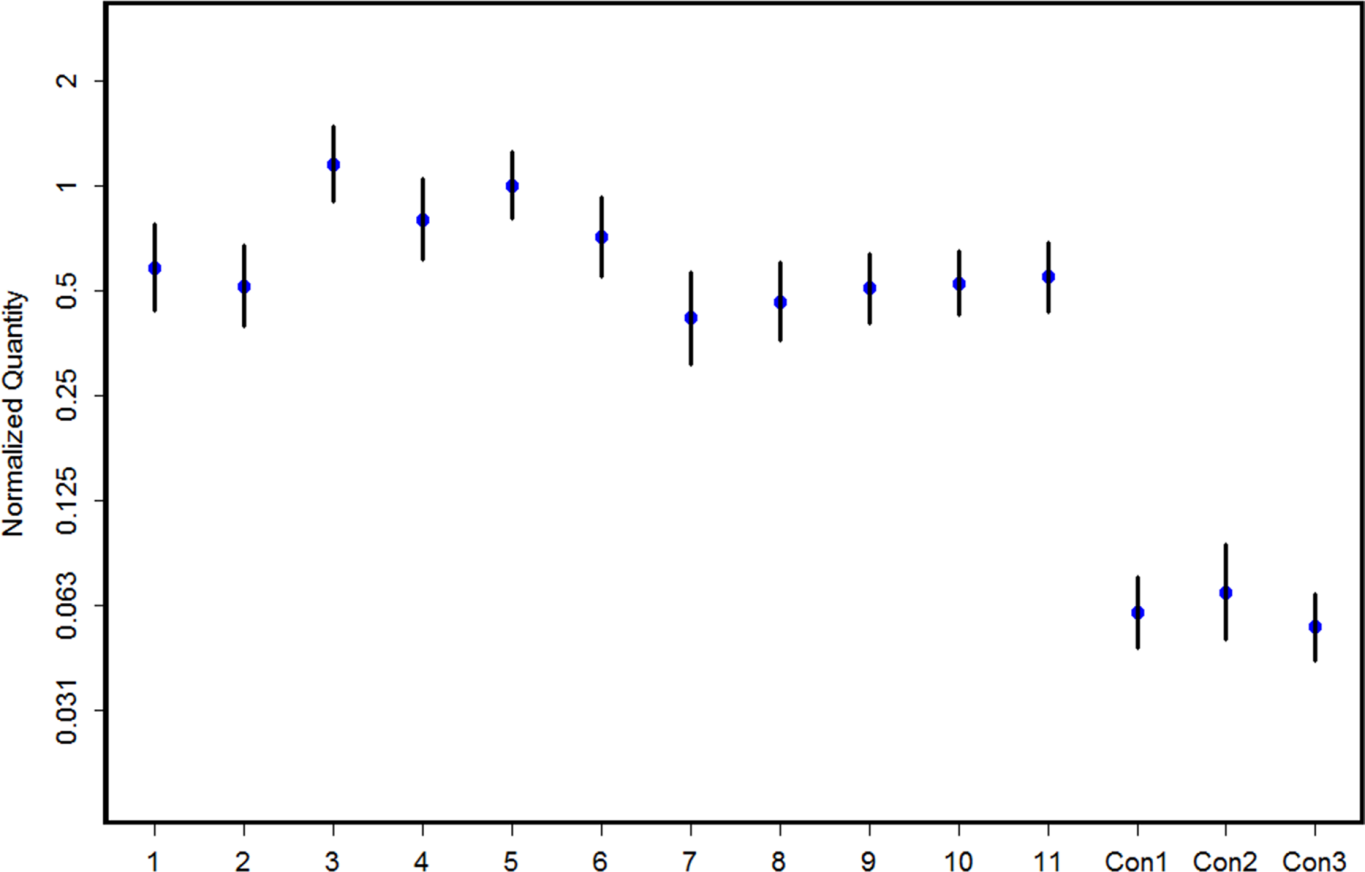
Quantification of transgene expression: 1 to 11 normalized quantity of the immunofluorescent signal of hair follicles normalized with the signal of chromaslides (i.e. the ratio between the two signals). Controls are on the right (Con1, Con2, Con3). Bars represent standard deviations of the log-ratio. Example of a six months old mouse on BDF_n_ genetic background

Sections of the dorsal skin of the transgenic lines used in this study were investigated following normalization to the fluorescence in the chromaslides. As shown by the example in Fig. 4, the expression of the reporter-gene was very stable in all samples investigated.

## Discussion

No in vivo experiments using transgenic mice have been performed so far that demonstrates the true role of the upstream regulatory region URR. The aim of this study was to learn more about the effect of the regulatory viral E2 protein on the modulation of the URR inHPV11 and whether the protein regulates transcription under in vivo conditions. It has been shown that the E2 protein plays a diverse and prominent role in the viral life cycle of human papillomaviruses. It is involved in the viral DNA replication, the segregation of viral genome into daughter cells as well as in the regulation of transcription. One particularly interesting aspect about E2 is that it can both activate and repress transcription, depending on numerous factors such as the cell type, position of E2-binding sites, as well as the nature and type of interaction of E2 polypeptides. Multiple in vitro experiments have been published showing that the E2 protein of high-risk papillomaviruses binds and modulates transcription of the URR (McBride 2013; Bouvard et al. 1994; Steger and Corbach 1997). However, the regulation of papillomaviruses is in general complex: For a complete upregulation of the promoter cellular factors are required, e.g. BRD-4, NF-1, AP-1, or AP-2 (Chong et al. 1990; Wu et al. 2016).

The human Ubiquitin C promoter regulates transcription of the transgenic E2. This leads to the ubiquitous expression of E2 (Leykauf et al. 2008) which was constant and not modulable (Schorpp et al. 1996). However, the occupancy of the four transcription factor binding sites and thus the repression or activation of the HPV-URR is largely dependent on the amount of E2 protein present (Sanders and Maitland 1994).

To date, the mouse is the only functional and established mammalian model in which human pathogenic viruses can be studied in vivo. Unlike in vitro, physiological processes are included.

### Modulation of URR activity through E2 protein

The data presented in this study demonstrate that the presence of the E2 protein modifies the expression of a URR11 reporter gene. The expression of the transgenic E2 protein was proven by Western blot analysis. (Fig. 2). A 42 kDa protein reacted with a specific anti-E2-antibody, this is the expected protein size (Antson et al. 2000). The slight double-band indicates possibly a partial phosphorylation. E2 exhibits phosphorylation sites, however, a possible phosphorylation was not studied in more detail. A partial phosphorylation argues also for the Janus-faced reactivity of E2 in vivo, both, upregulation and inhibition of the virus activity.

The presence of E2 leads to a higher portion of lacZ expressing hair follicles in combination with a greater statistical spread, this might be also due to a partial phosphorylation as discussed above. However, no additional tissue expressed the reporter gene as compared with the single transgenic URR11-lacZ mice. Thus, the presence of E2 does not alter cellular expression of the reporter gene and was only found in the same cell type as already observed in the single transgenic mice. This led to the conclusion that E2 protein acts in the end as an activator of URR in vivo*.* The high variability shown by statistical analyses reflects the different modulating properties of E2, i.e. up- and downregulation, however, up-regulation seems to be dominant. At the same time, HPV11-URR activity was also demonstrated by using antibodies against the reporter-gene and a detection with fluorescent dyes with and without the influence of E2 led to the same results. This strongly suggests that viral/epithelial cellular factors are needed for activation of the URR transcription activity and that these factors are exclusively localized in the epithelial hair cells (Tumbar et al. 2004). Histologically, these cells are identified as part of the bulge region of the hair, containing putative stem cells and being responsible for the reorganization of the epithelial layer of the skin.

The E2 protein can either activate or suppress transcription. E2 proteins are described to be unstable and are degraded by the proteasome, these parameters might contribute to the up- and downregulation of the URR-activity in the presence of E2 (Blachon et al. 2005). This study suggests that E2 is expressed in the skin and in the bulge of the hair, as well as in other tissues like the liver (Leykauf et al. 2008). These results therefore suggest that expression of both E2 and lacZ occur in the same cells, thus indicating that additional factors are required to modulate the expression of the URR. The finding that E1 is necessary for viral replication together with E2 suggests that both proteins play a role in transcription modulation (Berg and Stenlund 1997).

In an in vivo study, Cid et al. (1993) were able to modulate transcriptional activity of URR18-lacZ transgenic mice through treatment with dexamethasone. Unlike HPV18, HPV11 is classified as a low-risk papillomavirus with low oncogenic potential. In our findings treatments with dexamethasone or UV-radiation resulted in an enormous statistical spread without any significance. This underlines also the Janus-faced capacity of E2 to either induce or to repress the URR activity.

However, one has to keep in mind that this mouse model is in part artificial. Virus or natural host factors other than URR and E2 are not available, especially the partially conserved ORFs of the early protein E1, the two oncogenes E6 and E7 and the late genes L1 and L2, common in all HP viruses. E1 interacts with cellular replication factors. The regulatory N-terminal region is divergent, binds the cellular protein p80/UAF1 (USP associated factor 1) in HPV11 and HPV31 and is essential for replication in vivo (Bergvall et al. 2013). Also cellular factors are required for regulation, e.g. BRD-4, NF-1, AP-1, or AP-2 (Chong et al. 1990; Wu et al. 2016). Since these are in our model not species specific, the regulation of URR11 and E2 might be influenced. Nevertheless, our model gives deep insights into the role of E2 and the URR of HPV11.

### Age of mice

In the experiments performed by Cid et al. (1993), a sharp increase in the level of HPV18-URR driven transgene expression was found in newborn mice as compared to fetal tissues of the mice. In contrast, the intense URR-reactivity was not found in adult animals. This leads to the speculation that viral gene expression might be dependent on the age of the individual at the time of infection. However, investigating the HPV11-URR no significant age-related changes were detected. The transgenic E2 is under the control of the human Ubiquitin C-promoter (Schorpp et al. 1996). A transgenic gene construct using this promoter showed a reduced activity with the increasing age of the host (Passegué et al. 2001). However, this was not verified in this study.

### Genetic background of transgenic mice

Woodworth et al. (2004) have published that the induction of tumors in the presence of HPV16 was background dependent and showed in vitro and in vivo the highest induction rate on the FVB/N genetic background, up to ten-fold higher as compared to other genetic backgrounds. The reasons for these phenomena are complex and not fully understood. Similarly, several experimental studies on the role of HPV16 showed that the FVB/N strain is highly sensitive to skin carcinogenesis of mice (Herber et al. 1996; Lambert et al. 1993), as well as cervical cancer (Uc et al. 2020), possibly due to the availability of the E6/E7 proteins in the latter study. To elucidate if the FVB/N genetic background is more suitable for the modulation of the URR11 activity, the mice used in this study were back-crossed from their original B6D2F_n_ genetic background for two generations to FVB/N, resulting of a 75% portion of FVB/N in the genetic pool. The back-cross did not lead to dramatic changes but a trend towards a higher activity of the URR11 driven reporter-gene was detected. Since the studies investigating HPV16 on the FVB/N genetic background were in the presence of E6 and/or E7 which are definitely not present in our approach, E6 or E7 cannot be the only reason for this behavior. However, one has to take into account that HPV16 is a high-risk virus, whereas HPV11 investigated here is classified as a low-risk virus. It is possible, that the reduced aggressivity of HPV11 leads to a diminished virus activity. This might be due to the lack of the octamer-binding site of HPV11 found in the high-risk types (Morris et al. 1993).

### Quantification of expression

To quantify the expression of the transgene the relatively simple approach of using chromaslides was chosen. The advantage of chromaslides as the standard was that it allowed the comparison of the expression within a slide. Our first approach to use DAPI as a dye for standardization showed that DAPI does not stain the nuclei homogeneously; it therefore qualifies only for qualitative approaches, e.g. counterstains. Other investigations using fluorescent dyes to separate the transgene expression, cell membrane specific markers, a (DNA-) counterstain and the subsequently automated data analysis required (Kromp et al. 2020) have proven to be very complex and not suitable for the purposes of the study presented here.

Taken together, the presence of E2 in the transgenic URR11 model leads to an upregulation of the URR activity paralleled with a greater statistical spread, but the expression is very stable. The FVB/N genetic background seems to play a smaller role, also in case of the low risk HPV.
